# Preserving Patient‐Specific Knee Motion: A Randomized Clinical Trial of Unicompartmental and Total Knee Arthroplasty

**DOI:** 10.1002/jor.70077

**Published:** 2025-10-03

**Authors:** Gregor Kuntze, Sobhan Panjavi, Evonne Henning, Robert Korley, Gregory Abelseth, Janet Ronsky, Kelly Johnston

**Affiliations:** ^1^ Cumming School of Medicine University of Calgary Calgary Alberta Canada; ^2^ University of British Columbia British Columbia Canada; ^3^ McGill University Quebec Canada; ^4^ Schulich School of Engineering University of Calgary Calgary Alberta Canada

**Keywords:** gait biomechanics, knee arthroplasty, patient reported outcomes, randomized controlled trial

## Abstract

Unicompartmental knee arthroplasty (UKA) may enable improved functional outcomes compared to total knee arthroplasty (TKA). This randomized controlled trial assessed pre‐ and postoperative patient reported outcome measures (PROMs) and knee joint gait biomechanics for UKA and TKA patients. Patients were allocated to UKA (Oxford Partial Knee, Biomet, USA) and TKA (Persona CR Knee System, Zimmer, USA) study arms. Patients completed the Oxford Knee Score (OKS) and Western Ontario & McMaster University Arthritis Index (WOMAC), as well as instrumented gait analysis before and 1‐year after surgery. Measures of interest: OKS scores; WOMAC sub‐scores; Patient‐specific correlations and root mean squared errors (RMSE) of stance phase sagittal and coronal knee angles. Statistical analysis included linear mixed‐effects models (PROMs; *α* = 0.0125) and multivariate analysis of variance (gait biomechanics; *α* = 0.05). A total of 38 patients were recruited (UKA *n* = 17; TKA *n* = 21). All PROMs improved significantly following surgery (*n* = 37, *p* < 0.001), regardless of surgical technique. A significant effect of surgical technique on gait biomechanics was observed (*n* = 30, *F*_4,25_, *p* = 0.010), where UKA patients displayed greater sagittal plane correlations [median(Q1,Q3) UKA 0.985 (0.967, 0.991), TKA 0.955 (0.942, 0.973)]; *p* = 0.018] and lower coronal plane RMSEs [UKA 3.6 (2.4,5.0)°, TKA 8.6 (5.1, 11.5)°; *p* = 0.002]. Although patient‐reported outcomes improved similarly following UKA and TKA, UKA more closely preserved native knee kinematics as indicated by the greater similarity of sagittal gait patterns shapes and lower magnitude of coronal angle changes.

**Clinical Significance:**

Greater preservation of patient‐specific knee kinematics with UKA supports its use in appropriately selected patients and informs the design of targeted, functionally oriented rehabilitation protocols.

## 1 Introduction

Total knee arthroplasty (TKA) is one of the most common orthopedic surgical procedures to treat end‐stage knee osteoarthritis (OA) [1, 2]. However, despite the efficacy of TKA for mediating pain, up to 20% of patients are not fully satisfied with their TKA procedure [3, 4, 5, 6, 7]. This dissatisfaction presents an important clinical challenge, as the goals of surgery—pain relief, restoration of function, and improved quality of life —are not fully realized. Contributing factors to patient dissatisfaction following TKA include unmet expectations regarding postoperative outcomes, ongoing or residual pain, and persistent limitations in performing activities of daily living [8, 9]. Further potential contributors to patient dissatisfaction may include: impaired postoperative proprioception and balance, due to sacrifice of the anterior cruciate ligament (ACL) [10]; reduced knee extensor strength, which may affect functional recovery and the patient's ability to engage in physically demanding activities [11, 12]; and altered joint mechanics and muscle activations that may lead to abnormal gait and joint function [13, 14, 15].

While TKA is considered the gold‐standard, alternative surgical techniques, including unicompartmental knee arthroplasty (UKA), may offer advantages for a sub‐group of patients with isolated medial compartment knee OA. Specifically, UKA is a less invasive technique that preserves the patient's ACL and non‐arthritic lateral knee compartment, while replacing the medial compartment with a polyethylene insert. Current evidence indicates that, compared to TKA, UKA is associated with decreased infection rates, greater range of motion and joint function, and shorter hospital stays [16, 17]. Further, improvements in patient‐reported outcome measures (PROMs) [18] and gait function [19] have been reported for UKA patients. In this context, it may be suggested that UKA better preserves patient‐specific knee function. However, due to the use of predominantly cross‐sectional study designs, there is an unmet need for high‐quality research evidence on the longitudinal effects of UKA and TKA.

Therefore, the objective of this randomized controlled trial (RCT) was to assess the differences in pre‐ and postoperative PROMs and knee joint gait biomechanics for patients who received either a UKA or TKA. By addressing gaps in the current literature, this study aims to provide clinicians with evidence‐based guidance on the comparative functional differences between surgical techniques to inform clinical decision making and postoperative patient care.

## 2 Methods

### 2.1 Study Design

Single Center RCT (ClinicalTrials.gov ID# NCT02430129; Ethics ID# REB14‐0741). All data collections were performed between 2016 and 2021. Level of evidence I.

### 2.2 Participants

Patients were identified from four participating arthroplasty surgeons' new referral patient populations and recruited using consecutive sampling. Inclusion criteria were: anteromedial compartment OA; an intact ACL; normal, mild or moderate patellofemoral joint OA; angular deformity less than 15° (passively correctable to neutral); flexion contracture less than 5°; and age between 40 and 80 years old. Exclusion criteria were: severe patellofemoral joint OA or dislocation; history of past surgery on the affected knee, other than simple arthroscopy or open meniscectomy; inflammatory arthropathy; and a history of knee replacement or major ligamentous surgery of the contralateral knee. Patient eligibility, based on exclusion and inclusion criteria, was confirmed using the patient's medical history. Eligible patients were randomly allocated to either the UKA or TKA study arms using stratified randomization, where strata were based on patellofemoral joint OA (PFOA) involvement (i.e., no PFOA, mild/moderate PFOA). Randomization was performed by the study coordinator using the StudyTRAX (ScienceTrax, USA) online system.

### 2.3 Arthroplasty

All arthroplasties were performed by three fellowship‐trained arthroplasty surgeons (R.K., K.J., G.A.) using templating software to help choose implant size. UKAs were performed using the Oxford Partial Knee (Biomet, USA) and followed the recommended surgical approach. TKAs were performed via a medial parapatellar arthrotomy technique using the Persona CR Knee System (Zimmer, USA) without patellar resurfacing. All surgeons used gap balancing rather than measured resection for TKA. Conventional instrumentation was used in all TKA procedures. TKA cuts were made utilizing conventional instrumentation and mechanical alignment techniques, aiming for 5–6° of physiologic valgus.

### 2.4 Patient Reported Outcome Measures

Patients completed the Oxford Knee Score (OKS) [20] and Western Ontario and McMaster Universities Osteoarthritis Index (WOMAC) [21] questionnaires before surgery (baseline) and at three follow‐up time points (6‐weeks, 3‐months, 1‐year post surgery). The OKS is a 12‐item questionnaire for assessing function and pain after total knee arthroplasty. The OKS has demonstrated high reliability [20], and is among the best performing questionnaires for assessing the effects of joint arthroplasty [22]. The WOMAC is a self‐administered questionnaire of hip and knee pain, stiffness and function for patients with OA [21]. Reliability of the WOMAC has been reported as acceptable (pain and stiffness) and excellent (physical function), while test‐retest reliability has been reported as acceptable (pain and physical function), with weaker results for stiffness [23]. Use of WOMAC subscales has been recommended for investigations of knee arthroplasty outcomes [24].

### 2.5 Gait Assessment

Gait biomechanics were collected at baseline and 1‐year follow‐up. The same investigator (GK) performed all patient instrumentation [25]. Patients walked barefoot at a controlled pace (1.2 m/s ± 5%) along a raised walkway (8 m × 2 m). Gait kinematics and ground reaction forces (GRF) were recorded using a 10‐camera optical motion analysis system (Motion Analysis, USA) and two forceplates (AMTI, USA; Kistler Instrumente, CH). Patients were asked to walk along the center of the walkway while looking ahead towards the end of the walkway. Before data collections, patients performed a task familiarization to enable patients to achieve the target pace and determine their ideal starting position—that is, the initial placement that enabled patients to step on the forceplates without targeting. A starting position was determined at both ends of the walkway and gait data were then collected along both directions to minimize physical demands. A minimum of five successful walking trials were collected for each of the left and right legs. Successful walking trials consisted of walking at the specified pace and contact of the right or left leg within the boundaries of one of the forceplates.

### 2.6 Biomechanics Data Processing

Biomechanics data were computed using Visual3D (C‐Motion, USA) [25]. Joint angles were normalized to the stance phase of walking [i.e., heel strike (0%) to toe‐off (100%)]. Heel strike and toe‐off events were identified in Visual3D using the vertical GRF or a combination of the vertical GRF and the heel marker position.

Pearson correlation coefficient (r) and root mean square error (RMSE) analyses were used to assess the effects of UKA/TKA on knee joint kinematics of the operated leg. Correlation analysis enables the assessment of the linear relationship of sets of joint angle time‐series [26]—that is, the similarity of the shape of a patient's pre‐ and postoperative knee joint angles. Correlation coefficients were computed for patient‐specific mean stance phase time‐series (i.e., sagittal and coronal knee angles) using the corr function in Matlab (v2023b, MathWorks, USA):

(1) $$r=\frac{\sum \left(x_{i}-\bar{x}\right)(y_{i}-\bar{y})}{\sqrt{{\sum \left(x_{i}-\bar{x}\right)}^{2}{\sum \left(y_{i}-\bar{y}\right)}^{2}}}.$$

Where, *x_i_* and *y_i_* are the individual sample points and *x̄* and *ȳ* are the mean of the values respectively. The strength of r ranges from –1 to 1, indicating perfect negative or positive linear relationships respectively. A key advantage of correlation analysis is that it enables the comparison of joint angle time‐series, considering all data across the duration of stance‐phase, rather than relying on a priori gait event selection. However, it should be noted that the correlation analysis is not sensitive to the presence of constant data offsets (i.e., the magnitude of joint angles), where data linearity is maintained.

Due to the limitations of correlation analysis, differences in the magnitude of baseline and follow‐up knee joint angles (including constant offsets) were investigated by computing the RMSE for each participant (RMSE function, Matlab):

(2) $$\mathrm{RMSE}=\sqrt{\sum_{i=1}^{n}{\frac{\left(\hat{y}-y_{i}\right)}{n}}^{2}}.$$

Where, *ŷ* are the predicted values, *y_i_* are the observed values, and *n* are the number of observations. The RMSE indicates the average magnitude of the differences between data, where lower values indicate greater similarity and higher values indicate greater differences. The RMSE is sensitive to larger differences due to the squaring of differences, thereby placing greater weight on larger discrepancies.

### 2.7 Statistical Analysis

An a priori sample size estimation was performed using G*Power [27]. Based on OKS data by Walker et al. [28], who used a matched‐pairs design, the following model parameters were used: *F*‐test; ANOVA (repeated measures, within–between interaction); Cohen's *f* = 0.332; *α* = 0.05; power = 0.8; groups = 2; measurement points = 2; correlation among repeated measures = 0.6. This yielded a total sample size of 18 participants. Given that our research methods did not utilize a matched‐pairs design, the final sample size was doubled to account for the loss of within‐subject correlation. Including a predicted 10% drop‐out rate, the total sample size was determined to be 40 patients.

Statistical analyses were performed in R (v4.4.0, R Core Team, Austria). Effects of group and time‐since‐surgery on PROMs (i.e., OKS, WOMAC pain, stiffness, and physical function) were analyzed using separate linear mixed‐effects models using the lme function [29]. PROMs were considered as dependent variables, while group and time‐since‐surgery were considered as fixed effects. To account for repeated measures within patients, a random intercept for each patient study ID was included. Linear mixed effects models initially included interactions of group x time (Model A). If the interactions were not significant, interactions were removed, and the analysis was repeated (Model B). The consequence of model simplifications were assessed using likelihood ratio tests. Assumptions for normality of model residuals were assessed graphically using QQ plots and numerically using measures of skewness and kurtosis. To account for the use of multiple linear mixed‐effects models, the alpha level was adjusted using Bonferroni correction (*α* = 0.0125). Unfortunately, not all patients who completed the PROMs also participated in all biomechanics testing. Consequently, to ascertain the results, PROMs data analysis was repeated for patients participating in baseline and follow‐up gait assessment.

Group differences in stance phase sagittal and coronal knee joint angles (i.e., correlation coefficients and RMSEs) were assessed using multivariate analysis of variance (MANOVA function, *α* = 0.05) [30]. Data were first assessed for normality using Shapiro–Wilk tests. Correlation coefficients were then normalized using Fisher *Z*‐transformation and RMSE data were normalized using log transformation. If the MANOVA indicated a significant result, univariate ANOVAs were conducted for each dependent variable with Benjamini‐Hochberg adjustment to control the false discovery rate to determine the effects of group (TKA, UKA).

## 3 Results

### 3.1 Participants

A total of 38 patients were recruited (UKA *n *= 17, TKA *n* = 21). One UKA patient was excluded from the study due to revision surgery and conversion to TKA. PROMs responses were available for the remaining 37 patients (UKA *n* = 16, TKA *n* = 21), who completed all baseline and follow‐up questionnaires (Table 1).

**Table 1:** Patient demographics. Median (min, max).

	All patients		Patients with PROMs		Patients with gait assessment	
	UKA	TKA	UKA	TKA	UKA	TKA
*n*	17	21	16	21	12	18
Age, Yrs	61 (45, 75)	66 (51, 77)	60 (45, 75)	66 (51, 77)	61 (45, 75)	66 (51, 77)
Sex, *n*	M 5, F 12	M 8, F 13	M 5, F 11	M 8, F 13	M 5, F 7	M 8, F 10
Height, m	1.65 (1.52, 1.86)	1.67 (1.47, 1.86)	1.66 (1.52, 1.86)	1.67 (1.47, 1.86)	1.67 (1.52, 1.86)	1.67 (1.52, 1.86)
Weight, kg	92.5 (68.0, 130.1)	93.0 (54.0, 144.0)	92.7 (68.0, 130.1)	93.0 (54.0, 144.0)	92.7 (68.0, 101.5)	93.0 (72.5, 144.0)
BMI	32.8 (23.7, 52.2)	33.2 (23.4, 44.7)	32.2 (23.7, 52.2)	33.2 (23.4, 44.7)	31.0 (25.3, 40.1)	32.9 (26.9, 44.7)

One UKA patient was excluded due to challenges regarding high BMI (> 50) with conventional, marker‐based, gait analysis. Due to concerns over COVID, one UKA patient did not attend gait assessment and four patients (UKA 1, TKA 3) were lost to follow‐up. Three patients (UKA *n* = 2, TKA *n* = 1) were unable to walk at the required pace (1.2 m/s ± 5%) at baseline. However, for two of these patients (UKA *n* = 1, TKA *n* = 1), follow‐up data were available where the patients matched their slower baseline walking pace. The slow pace‐matched data were then included in the analysis, while the participant without pace‐matched data was excluded. Therefore, baseline and follow‐up biomechanics data were available for a total of 30 patients (UKA *n* = 12, TKA *n* = 18).

### 3.2 Patient Reported Outcome Measures

**Figure 1:**
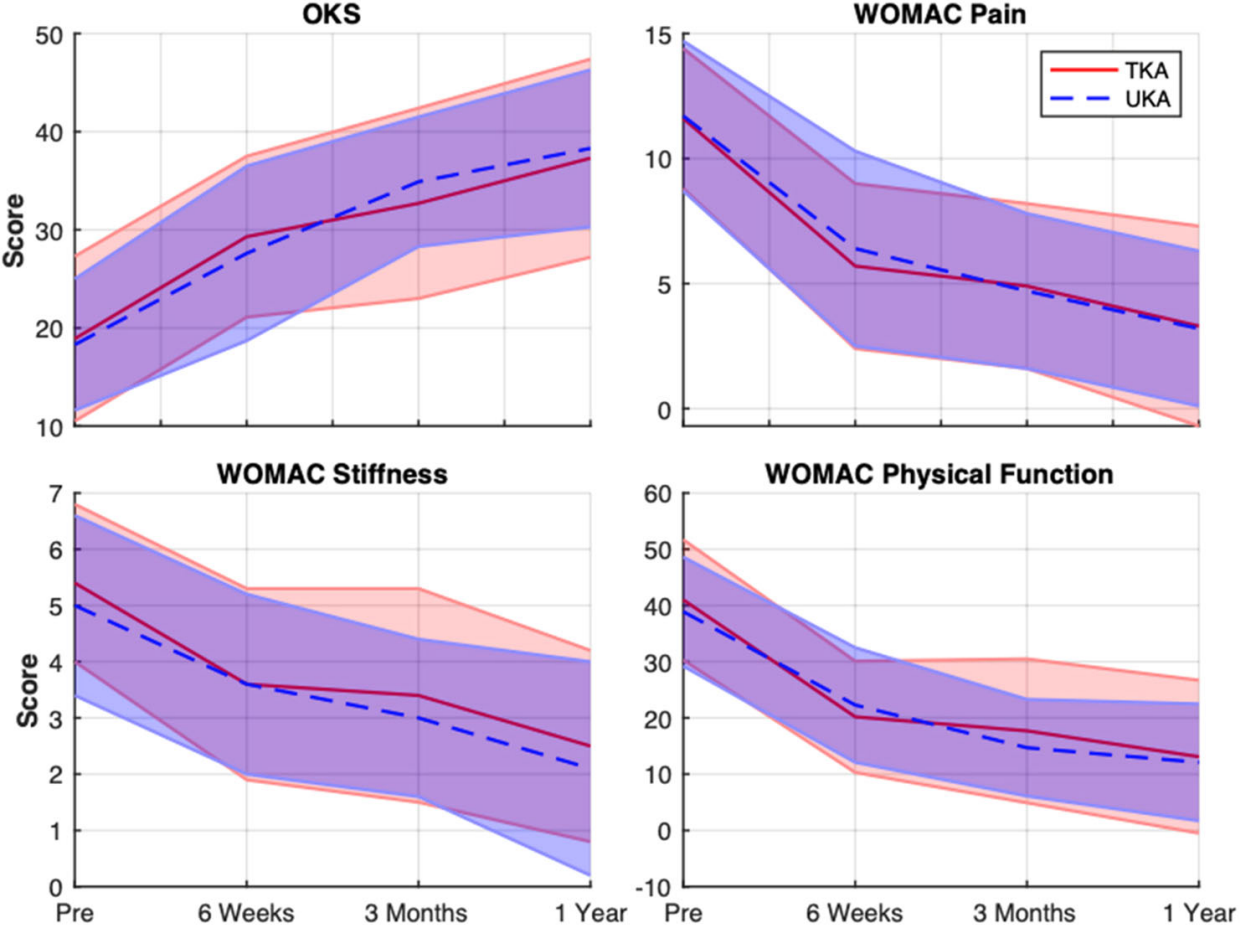
Changes in OKS and WOMAC responses for all UKA and TKA patients across follow‐up periods. Data are presented as means and standard deviations.

No significant interactions between group and time‐since‐surgery (Model A; Table 2, Figure 1) were observed. Therefore, surgical technique did not appear to affect PROMs responses for UKA and TKA patients in this cohort. Model B (excluding interactions) revealed a significant effect of time‐since‐surgery on all PROMs (i.e., *p* < 0.001 for all PROMs), where PROMs indicated significant improvements at all follow‐up time points compared to baseline. Repeated analysis, for the 30 patients who participated in all gait assessment, indicated similar responses with regard to P‐values and magnitudes of the beta coefficients and 98.75% confidence intervals (Table 3).

**Table 2:** PROMs Results (*n* = 37): Linear Mixed‐Effects Estimates (*β*, 98.75% CI, *p*) for Time Effects and Group × Time Interactions ‐ Model A (with interactions) and Model B (Main Effects).

PROMs		Model A		Model B	
Fixed effects & interactions		*β* (98.75% CI)	*p*	*β* (98.75% CI)	*p*
WOMAC Pain	6 Weeks	−5.3 (−7.4 to −3.2)	< 0.001*	−5.5 (−7.1 to −4.0)	< 0.001*
	3 Months	−7.0 (−9.2 to −4.9)	< 0.001*	−6.9 (−8.4 to −5.3)	< 0.001*
	1 Year	−8.5 (−10.6 to −6.4)	< 0.001*	−8.4 (−10.0 to −6.8)	< 0.001*
	Group × 6 Weeks	−0.6 (−3.8 to 2.6)	0.642	—	—
	Group × 3 Months	0.4 (−2.8 to 3.6)	0.738	—	—
	Group x 1 Year	0.3 (−2.9 to 3.5)	0.829	—	—
WOMAC Stiffness	6 Weeks	−1.5 (−2.5 to −0.5)	< 0.001*	−1.6 (−2.3 to −0.8)	< 0.001*
	3 Months	−2.0 (−3.0 to −1.0)	< 0.001*	−2.8 (−2.0 to −1.3)	< 0.001*
	1 Year	−3.0 (−4.0 to −2.0)	< 0.001*	−2.9 (−3.7 to −2.2)	< 0.001*
	Group × 6 Weeks	−0.3 (−1.8 to 1.2)	0.649	—	—
	Group × 3 Months	0.0 (−1.5 to 1.6)	0.937	—	—
	Group × 1 Year	0.1 (−1.4 to 1.6)	0.898	—	—
WOMAC Physical Function	6 Weeks	−16.5 (−23.6 to −9.5)	< 0.001*	−18.4 (−23.7 to −13.1)	< 0.001*
	3 Months	−24.2 (−31.2 to −17.1)	< 0.001*	−23.8 (−29.1 to −18.5)	< 0.001*
	1 Year	−26.8 (−33.8 to −19.7)	< 0.001*	−27.2 (−32.5 to −22.0)	< 0.001*
	Group × 6 Weeks	−4.3 (−15.0 to 6.4)	0.312	—	—
	Group × 3 Months	0.9 (−9.8 to 11.6)	0.836	—	—
	Group × 1 Year	−1.1 (−11.8 to 9.6)	0.793	—	—
OKS	6 Weeks	9.3 (4.3 to 14.2)	< 0.001*	9.8 (6.1 to 13.5)	< 0.001*
	3 Months	16.5 (11.6 to 21.5)	< 0.001*	15.3 (11.6 to 19.0)	< 0.001*
	1 Year	20.0 (15.0 to 24.9)	< 0.001*	19.3 (15.6 to 23.0)	< 0.001*
	Group × 6 Weeks	1.1 (−6.4 to 8.6)	0.713	—	—
	Group × 3 Months	−2.8 (−10.3 to 4.8)	0.350	—	—
	Group × 1 Year	−1.6 (−9.1 to 5.9)	0.595	—	—

* Indicates a significant effect of time since surgery.

**Table 3:** PROMs results (*n* = 30): linear mixed‐effects estimates (*β*, 98.75% CI, *p*) for Time effects and group × time interactions—Model A (with interactions) and Model B (Main Effects).

PROMs		Model A		Model B	
Fixed effects & interactions		*β* (98.75% CI)	*p*	β (98.75% CI)	*p*
WOMAC pain	6 Weeks	−5.7 (−7.9 to −3.6)	< 0.001*	−5.8 (−7.5 to −4.2)	< 0.001*
	3 Months	−7.2 (−9.3 to −5.0)	< 0.001*	−7.0 (−8.7 to −5.4)	< 0.001*
	1 Year	−8.8 (−10.9 to −6.6)	< 0.001*	−8.8 (−10.4 to −7.2)	< 0.001*
	Group × 6 Weeks	−0.3 (−3.7 to 3.1)	0.835	—	—
	Group × 3 Months	0.3 (−3.1 to 3.7)	0.802	—	—
	Group × 1 Year	−0.1 (−3.4 to 3.3)	0.967	—	—
WOMAC stiffness	6 Weeks	−1.6 (−2.7 to −0.5)	< 0.001*	−1.7 (−2.5 to −0.8)	< 0.001*
	3 Months	−2.1 (−3.2 to −0.9)	< 0.001*	−2.1 (−3.0 to −1.3))	< 0.001*
	1 Year	−3.0 (−4.1 to −1.9)	< 0.001*	−3.0 (−3.9 to −2.2)	< 0.001*
	Group × 6 Weeks	−0.1 (−1.9 to 1.6)	0.840	—	—
	Group × 3 Months	−0.2 (−1.9 to 1.6)	0.778	—	—
	Group × 1 Year	−0.1 (−1.8 to 1.7)	0.904	—	—
WOMAC physical function	6 Weeks	−17.8 (−24.9 to −10.8)	< 0.001*	−18.9 (−24.3 to −13.5)	< 0.001*
	3 Months	−25.0 (−32.0 to −18.0)	< 0.001*	−24.8 (−30.1 to −19.4	< 0.001*
	1 Year	−27.3 (−34.4 to −20.3)	< 0.001*	−28.3 (−33.7 to −22.9)	< 0.001*
	Group × 6 Weeks	−2.7 (−13.8 to 8.4)	0.542	—	—
	Group × 3 Months	0.6 (−10.5 to 11.7)	0.894	—	—
	Group × 1 Year	−2.4 (−13.5 to 8.7)	0.580	—	—
OKS	6 Weeks	10.8 (5.9 to 15.7)	< 0.001*	10.5 (6.8 to 14.3)	< 0.001*
	3 Months	16.9 (12.1 to 21.8)	< 0.001*	16.3 (12.6 to 20.0)	< 0.001*
	1 Year	20.6 (15.7 to 25.4)	< 0.001*	20.5 (16.7 to 24.2)	< 0.001*
	Group × 6 Weeks	−0.6 (−8.3 to 7.1)	0.841	—	—
	Group × 3 Months	−1.6 (−9.3 to 6.1)	0.596	—	—
	Group × 1 Year	−0.2 (−8.0 to 7.5)	0.942	—	—

* Indicates a significant effect of time since surgery.

### 3.3 Gait Biomechanics

**Figure 2:**
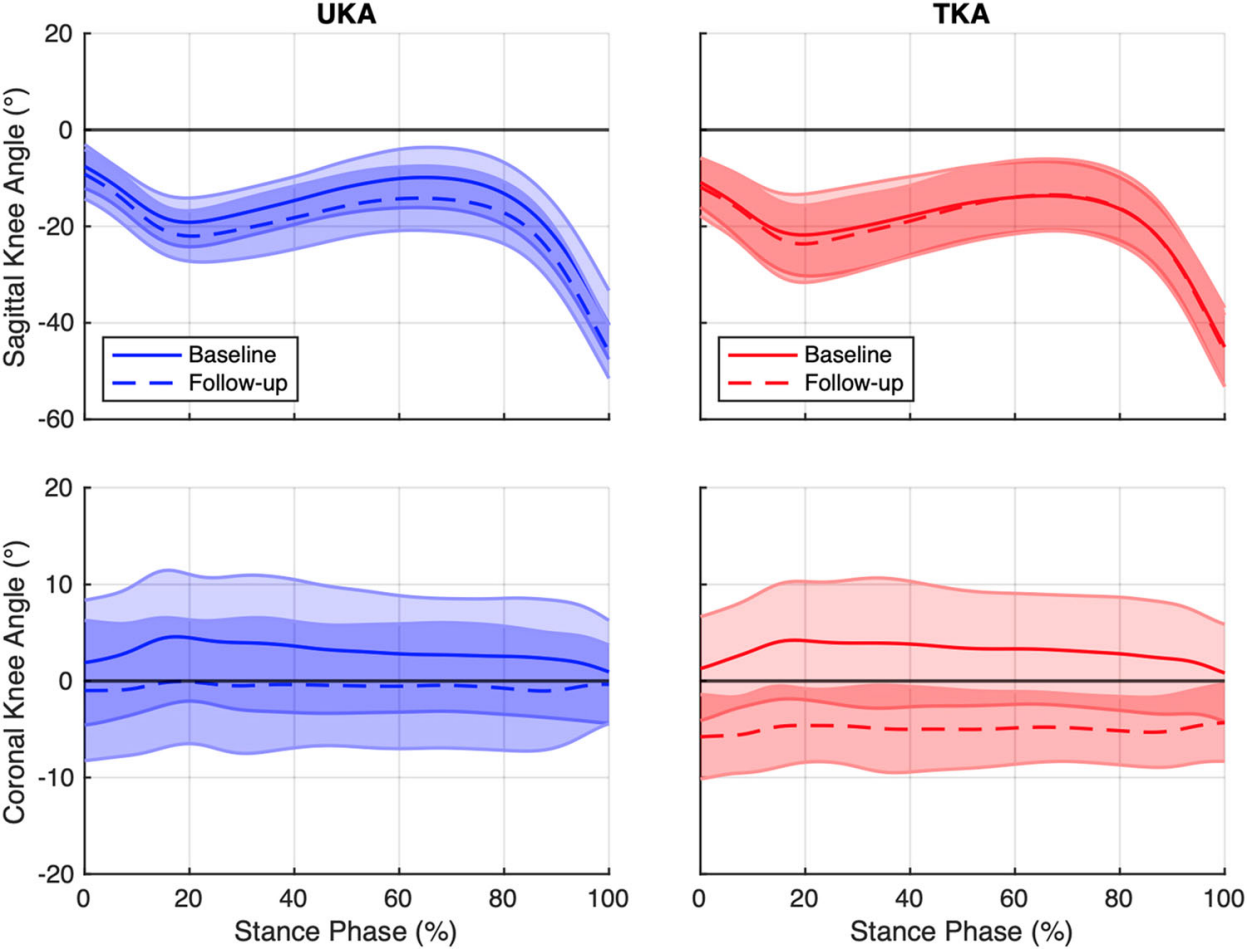
Pre‐ and postoperative gait biomechanics for UKA and TKA patients. Sagittal and coronal knee angles at baseline and follow‐up, as well as patient‐specific changes (follow‐up‐baseline), are presented as means and standard deviations. Data are normalized to the stance phase of gait starting at heel strike (0%) and ending at toe‐off (100%). Positive values represent knee extension and adduction (i.e., varus).

**Figure 3:**
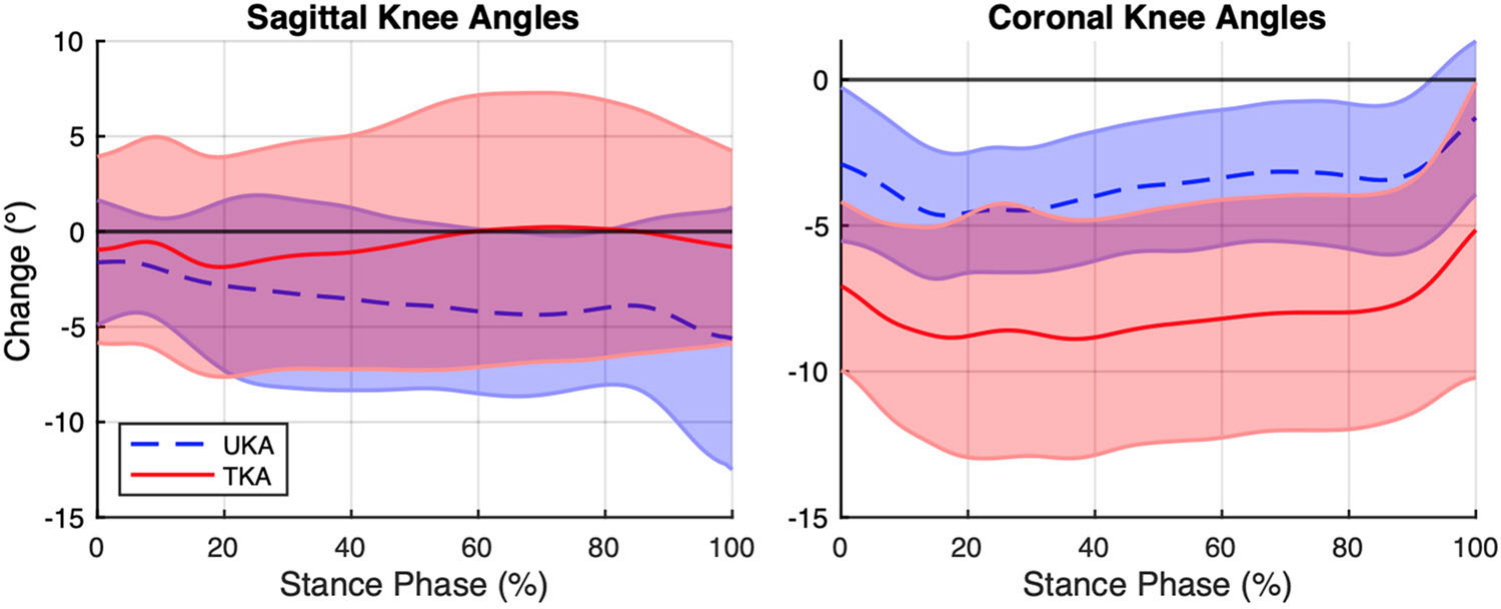
Mean within‐patient changes in sagittal and coronal knee angles (Follow‐up – Baseline) for UKA and TKA patients. Positive values represent knee extension and adduction (i.e., varus).

A significant effect of surgical technique was observed for the combination of correlation and RMSE data for sagittal and coronal knee angles of the operated knee (MANOVA; *F*_4,25_, *p *= 0.010; Table 4, Figure 2). Follow‐up testing using univariate ANOVAs revealed that correlations of baseline and follow‐up sagittal plane knee angles were significantly higher for UKA patients than TKA patients [median(Q1,Q3) UKA 0.985 (0.967, 0.991), TKA 0.955 (0.942, 0.973); *p* = 0.018] (Figure 3). Therefore, the shapes of sagittal knee angle curves at follow‐up were more similar to their respective baselines for UKA compared to TKA patients. Conversely, TKA patients appeared to demonstrate a larger variance of patient‐specific changes in sagittal knee angles for most of stance compared to UKA patients (Figure 3). In contrast, surgical technique did not appear to affect correlations of baseline and follow‐up knee angles in the coronal plane (*p* = 0.283).

**Table 4:** Summary of knee joint gait biomechanics measures.

		Group	Median	(Min, Max)	IQR (Q1, Q3)	
Correlations	Sagittal plane	UKA	0.985	(0.937, 0.998)	(0.967, 0.991)	*
		TKA	0.955	(0.820, 0.997)	(0.942, 0.973)	
	Coronal plane	UKA	0.675	(−0.632, 0.927)	(0.526, 0.848)	
		TKA	0.383	(−0.319, 0.974)	(0.192, 0.633)	
RMSE (deg)	Sagittal plane	UKA	4.3	(1.0, 10.5)	(2.2, 5.8)	
		TKA	3.9	(1.5, 12.5)	(3.0, 6.2)	
	Coronal plane	UKA	3.6	(1.4, 7.4)	(2.4, 5.0)	*
		TKA	8.6	(2.1, 14.0)	(5.1, 11.5)	

Abbreviations: IQR, interquartile range; RMSE, root mean square error; TKA, total knee arthroplasty; UKA, unicompartmental knee arthroplasty.

* Indicates significant differences for normalized data included in the MANOVA model.

Regarding the magnitude of differences in joint angles between baseline and follow‐up (Figure 3), UKA patients displayed significantly lower RMSE values for coronal plane knee angles than TKA patients [UKA 3.6 (2.4, 5.0)°, TKA 8.6 (5.1, 11.5)°; *p* = 0.002]. Therefore, UKA patients experienced smaller changes than TKA patients in dynamic joint alignment in the coronal plane. However, no significant effect of surgical technique was observed for sagittal plane knee angles (*p* = 0.653).

## 4 Discussion

The findings indicate that, regardless of surgical technique, patients reported significant improvements in PROMs at all follow‐up time points compared to their pre‐surgical baseline. Importantly, UKA patients displayed knee joint kinematics profiles that more closely resembled their native knee motions at the 1‐year post‐surgery assessment than TKA patients. These findings demonstrate the differential effects of surgical technique on dynamic joint function. Further research is needed to better delineate the time course of postoperative recovery and inform patient‐specific follow‐up and rehabilitation strategies.

Contrary to current evidence [31], no differences in PROMs scores were observed between UKA and TKA patients (Table 2, Table 3). It should be acknowledged that due to recruitment and follow‐up challenges, related in part to COVID, the desired sample size of 40 patients, needed to discern differences in OKS scores between groups, was not achieved. It is therefore feasible that differences in surgical technique may be observed for a larger sample of patients. However, fortunately, all patients demonstrated clinically significant improvements [32, 33] in OKS scores, as well as all WOMAC sub‐scores (Figure 1). Therefore, knee joint arthroplasty provided satisfactory outcomes for both UKA and TKA patients. An interesting observation is that UKA has been associated with shorter functional recovery [34]. Unfortunately, measures of the time course of recovery were not included in this study. Nevertheless, it may be speculated that, given the observed differences in biomechanical responses, UKA patients in this cohort would also have experienced faster postsurgical recovery. While supporting evidence is not available, the time course of recovery is a continuing topic of interest and alternative PROMs such as the forgotten joint score [35] may be beneficial to more clearly delineate the effects of joint arthroplasty technique on postoperative recovery and patient satisfaction.

Despite a lack of evidence for a significant effect of arthroplasty technique on PROMs for this cohort, surgical technique did appear to have a significant effect on postsurgical knee function. Specifically, UKA appeared to more closely retain the patients' native knee mechanics, akin to the surgical goals of kinematic alignment. Kinematic alignment refers to the positioning of the prosthetic components in such a way as to mimic the patient′s natural knee alignment and joint motion. The potential advantage of this approach relates to improved postoperative joint function [36] and resultant improved patient satisfaction. While kinematic alignment was not a primary objective of either UKA or TKA techniques, UKA retains more of the patient′s original anatomy and alignment, promoting the restoration of native knee mechanics.

Evidence for the preservation of patient‐specific knee motions with UKA vs TKA was observed both in the shape of sagittal knee angles and the magnitude of coronal knee angles. Specifically, stronger correlations of sagittal knee angles demonstrate that UKA patients walked with more similar knee excursion patterns at 1‐year follow‐up compared to their baseline (Table 4). Visualizing the mean within‐patient changes in sagittal knee angles (Figure 3) demonstrates that TKA patients displayed greater variability in kinematic changes, particularly during the loading response (~0%–20% stance) and terminal stance phases (~50%–80% stance). Therefore, TKA patients appeared to display more variable postoperative sagittal knee movement patterns with some patients walking with greater knee flexion and others with greater knee extension. In contrast, UKA postoperative patterns appeared to more closely reflect preoperative kinematic patterns. To the best knowledge of the authors, these findings are unique observations, not previously reported for joint arthroplasty. It is feasible that more variable knee kinematics responses could contribute to reports of slower functional recovery for some TKA patients compared to the faster functional recovery reported for UKA patients [34].

Greater preservation of patient‐specific knee motions was further reflected in the more neutral joint alignment of UKA patients in the coronal plane (Figure 2, Table 4). Specifically, UKA patients displayed a smaller magnitude of change following surgery (Figure 3), with a coronal alignment close to zero across patients at follow‐up. In contrast, TKA patients were in greater dynamic valgus, as reported by Agarwal et al. [37], and appeared to display a more variable range of coronal knee angles (Figure 3). It is feasible that this larger variability following TKA may be a limitation of conventional instrumentation used in this study and may be lessened by new technologies such as robotic‐assistance. In contrast, no differences in overall joint angle magnitudes were observed in the sagittal plane, supporting the findings of a systematic review by Dong et al. [19]. Therefore, while the shape (i.e., correlation) of sagittal knee angles during stance phase appeared to differ between groups, the magnitude of the change (i.e., RMSE) of sagittal knee angles across stance appeared to be comparable between groups.

### 4.1 Strengths and Limitations

This study implemented a strong research design, minimizing selection bias and enabling insight into longitudinal responses to knee arthroplasty. The main study limitations relate to the relatively small sample size and single‐center setting, which affect the generalizability of the study findings. It should be acknowledged that the WOMAC is prone to a high correlation between pain and physical function sub‐scales whereby pain is pain with function rather than pain in its own right. Further, the results of this study may have been affected by potential confounders including patient sex, rehabilitation participation, and contralateral knee conditions. Larger multicenter trials are needed to adequately account for such confounders using statistical models. Interpretation of joint biomechanics responses may differ from previous literature due to the control of walking pace. Differences in gait velocity may affect joint excursion magnitudes, thereby potentially introducing changes not related to or masking the effects of joint arthroplasty. Consequently, it was deemed that controlling walking pace was a preferred methodological approach to support the research objectives.

### 4.2 Significance

These findings contribute to a growing body of evidence suggesting that UKA should be offered to more patients, when appropriate, challenging the traditional preference for TKA. Greater preservation of patient‐specific knee kinematics with UKA may support faster functional recovery. Further research is needed to investigate the time‐course of arthroplasty‐specific biomechanical adaptations to inform the planning of post‐surgery follow‐up and support the design of targeted, functionally oriented rehabilitation protocols.

## Author Contributions

**Gregor Kuntze:** research design, data acquisition, analysis and interpretation; drafting and critically revising the paper. **Sobhan Panjavi:** data analysis and interpretation, critically revising the paper. **Evonne Henning:** data analysis and interpretation, critically revising the paper. **Robert Korley:** research design, data interpretation, critically revising the paper. **Gregory Abelseth:** research design, data interpretation, critically revising the paper. **Janet Ronsky:** research design, data interpretation, critically revising the paper. **Kelly Johnston:** research design, data interpretation, critically revising the paper. All authors have read and approved the final submitted manuscript.
